# A link between central kynurenine metabolism and bone strength in rats with chronic kidney disease

**DOI:** 10.7717/peerj.3199

**Published:** 2017-04-20

**Authors:** Bartlomiej Kalaska, Krystyna Pawlak, Ewa Oksztulska-Kolanek, Tomasz Domaniewski, Beata Znorko, Malgorzata Karbowska, Aleksandra Citkowska, Joanna Rogalska, Alicja Roszczenko, Malgorzata M. Brzoska, Dariusz Pawlak

**Affiliations:** 1 Department of Pharmacodynamics, Medical University of Bialystok, Bialystok, Poland; 2 Department of Monitored Pharmacotherapy, Medical University of Bialystok, Bialystok, Poland; 3 Department of Toxicology, Medical University of Bialystok, Bialystok, Poland

**Keywords:** Bone disorders, Chronic kidney disease, Kynurenine pathway, Brain

## Abstract

**Background:**

Disturbances in mineral and bone metabolism represent one of the most complex complications of chronic kidney disease (CKD). Serotonin, a monoamine synthesized from tryptophan, may play a potential role in bone metabolism. Brain-derived serotonin exerts a positive effect on the bone structure by limiting bone resorption and enhancing bone formation. Tryptophan is the precursor not only to the serotonin but also and primarily to kynurenine metabolites. The ultimate aim of the present study was to determine the association between central kynurenine metabolism and biomechanical as well as geometrical properties of bone in the experimental model of the early stage of CKD.

**Methods:**

Thirty-three Wistar rats were randomly divided into two groups (sham-operated and subtotal nephrectomized animals). Three months after surgery, serum samples were obtained for the determination of biochemical parameters, bone turnover biomarkers, and kynurenine pathway metabolites; tibias were collected for bone biomechanical, bone geometrical, and bone mass density analysis; brains were removed and divided into five regions for the determination of kynurenine pathway metabolites.

**Results:**

Subtotal nephrectomized rats presented higher serum concentrations of creatinine, urea nitrogen, and parathyroid hormone, and developed hypocalcemia. Several biomechanical and geometrical parameters were significantly elevated in rats with experimentally induced CKD. Subtotal nephrectomized rats presented significantly higher kynurenine concentrations and kynurenine/tryptophan ratio and significantly lower tryptophan levels in all studied parts of the brain. Kynurenine in the frontal cortex and tryptophan in the hypothalamus and striatum correlated positively with the main parameters of bone biomechanics and bone geometry.

**Discussion:**

In addition to the complex mineral, hormone, and metabolite changes, intensified central kynurenine turnover may play an important role in the development of bone changes in the course of CKD.

## Introduction

Abnormal mineral, endocrine, and bone metabolism represents one of the most complex complications of chronic kidney disease (CKD). In patients with CKD, the kidneys fail to excrete a phosphate appropriately, leading to hyperphosphatemia and decreased biologically active form of vitamin D. Vitamin D deficiency causes a reduction in intestinal calcium absorption and increase in parathyroid hormone (PTH) concentration with associated elevations in the levels of fibroblast growth factor-23. This systemic disorder, commonly known as CKD-mineral and bone disorder (CKD-MBD), can be manifested by decreased quality of life and increased fractures, morbidity, and mortality (Moe et al., 2006; Moe et al., 2009). Although the relationships between CKD and bone disturbances have been studied for many years, the exact pathophysiology of CKD-MBD remains unclear.

The neurotransmitter regulation may be involved in the development of CKD-MBD (Wu et al., 2015). Although many brain-derived neurotransmitters do not cross the blood–brain barrier, they may act on bone metabolism through an indirect mechanism. Indeed, the brain-derived serotonin does not cross the blood–brain barrier (Mann et al., 1992) and yet it exerts a positive effect on the bone structure by limiting bone resorption and enhancing bone formation. Serotonin as a neurotransmitter acts on neurons of the ventromedial hypothalamic nuclei, activates serotonin receptor 5-HT_2C_, decreases sympathetic tone and thereby supports bone mass density (Yadav et al., 2009). Interestingly, serotonin, when produced peripherally, exerts opposite influences on the bone formation (Yadav et al., 2008; Ducy & Karsenty, 2010). Our recent correlative evidence confirms that the elevated peripheral serotonin may adversely affect the strength and metabolism of long bones in rats with experimental CKD (Pawlak et al., 2016).

Both centrally and peripherally produced serotonin is synthesized from the precursor tryptophan (TRP) by two distinct enzymes (O’Mahony et al., 2015). TRP is the precursor not only to the serotonin but also and primarily to kynurenine metabolites (Schwarcz, 2004). Kynurenine pathway plays a crucial role in several processes, including redox homeostasis (Gonzalez Esquivel et al., 2017), gluconeogenesis (Dayer, Safari & Dayer, 2009), diabetic retinopathy (Munipally et al., 2011), inflammation (Heyes et al., 1992), carcinogenesis (Prendergast, 2011), and apoptosis (Fallarino et al., 2002). The knowledge of the role of the kynurenine pathway in bone metabolism is limited. Bone mineral density was associated with several of the kynurenines (Apalset et al., 2014). Patients with osteoporosis presented lower levels of TRP and 3-hydroxyanthranilic acid, whereas higher levels of anthranilic acid compared with healthy controls (Forrest et al., 2006). Moreover, TRP degradation *via* kynurenine pathway was also increased during osteoblastogenesis (Vidal et al., 2015). We have previously demonstrated the serious behavioral and severe central kynurenine pathway disturbances in rats with end-stage chronic renal insufficiency (Topczewska-Bruns et al., 2001; Topczewska-Bruns et al., 2002). The ultimate aim of the present study was to determine the association between central kynurenine pathway metabolites and biomechanical as well as geometrical properties of bone in an experimental model of CKD in rats.

## Materials & Methods

### Animals

Wistar rats were purchased from the Center of Experimental Medicine in Medical University of Bialystok. Rats were housed in temperature and humidity controlled room according to Good Laboratory Practice rules. They were allowed to have ad libitum access to sterilized tap water and standard chow (Ssniff R-Z V1324). All procedures involving animals were approved by Local Ethical Committee on Animal Testing at the Medical University of Bialystok (Permit Number 17/2012) and conducted by ARRIVE guidelines (Kilkenny et al., 2010), EU Directive 2010/63/EU for animal experiments and the Council on the protection of animals used for scientific purposes.

### Design of experiment

Thirty-three Wistar rats weighing 117 ± 16 g were randomly divided into two groups: sham-operated (Sham, *n* = 15) and subtotal nephrectomized rats (5/6 Nx, *n* = 18). The subtotal nephrectomy was performed according to the procedure described by Sviglerova et al. (2010). Sham-operated rats underwent renal evacuation and decapsulation. Three months after surgery, rats were weighed and anesthetized intraperitoneally with ketamine (100 mg/kg) and xylazine (10 mg/kg). Blood samples were taken from the heart and centrifuged to obtain serum for 10 min at 4,000× g. After centrifugation serum was frozen until biochemical and high-performance liquid chromatography (HPLC) analysis. Then, tibias were dissected, cleaned of adhering soft tissue, weighted using electronic scales Kern ALT 100-5-A (Kern, Bellingen, Germany), measured with calipers (Artpol, Warszawska, Poland), and frozen until biomechanical and geometrical analysis. Brains were removed, divided into five regions (cerebellum, brainstem, frontal cortex, hypothalamus, striatum), immediately frozen and stored at −80 °C until HPLC analysis.

### Serum biochemistry

Serum urea and creatinine concentrations were measured using automated biochemical analyzer (Mindray BS-120; Mindray, Mahwah, NJ, USA) with the commercially available kit (CORMAY, Poland). Serum inorganic phosphorus, calcium, and alkaline phosphatase (ALP) were measured using commercially available kits (BioMaxima, Lublin, Poland). Intact parathyroid hormone (PTH) osteoclast-derived tartrate-resistant acid phosphatase form 5b (TRACP 5b) were determined by ELISA using commercially available colorimetric kits purchased from Immunotopic (USA) and Immunodiagnostic Systems (Frankfurt am Main, Germany), respectively.

### Bone biomechanics

Before biomechanical analysis, tibias were thoroughly thawed to room temperature. Bone mechanical properties were determined using the three-point bending test as described previously (Brzoska, Majewska & Moniuszko-Jakoniuk, 2005). The testing was performed using machine Zwick Roell Z.2.5 (Zwick, Stuttgart, Germany). Bone mechanical parameters included stiffness, the resistance of the tibia diaphysis to deformation (yield load), the resistance of the tibia diaphysis to fracture (ultimate load), their displacements, and work to fracture (Oksztulska-Kolanek et al., 2016).

### Cross-sectional geometry and bone mass density

Bone fragments obtained after biomechanical analysis were measured with calipers to obtain geometrical parameters: anterior-posterior periosteal diameter, anterior-posterior endosteal diameter, medial-lateral periosteal diameter, medial-lateral endosteal diameter, and wall thickness. Cortical index, cross-sectional area, mean relative wall thickness and cross-sectional moment of inertia were calculated using formulas described previously (Brzoska, Majewska & Moniuszko-Jakoniuk, 2005; Gajos-Michniewicz et al., 2012). Archimedes’ principle determination of bone density was calculated using the formula described by Keenan et al. (1997).

### HPLC analysis

Serum and brain concentrations of TRP, kynurenine (KYN), and 3-hydroxykynurenine (3HK) were determined by HPLC (Agilent 1260 series; Agilent Technologies, Santa Clara, CA, USA). Deproteinized serum samples were prepared by adding 2 M perchloric acid. Samples were vortexed, kept at 4 °C for 10 min, and centrifuged at 14,000× g for 30 min at 4 °C. The supernatant was injected into HPLC system for analysis. Brain tissues were homogenized in 20% trichloroacetic acid containing 0.1% EDTA. The samples were centrifuged at 14,000× g for 20 min at 4 °C. After centrifugation, the supernatant was filtered (0.45 µm Millipore filter) and stored at −80 °C until assayed.

TRP and KYN concentrations were measured according to Holmes (1988). The prepared samples (2 µL) were separated on ODS column (Waters Spherisorb 3 µm ODS 2, 2.1 × 150 mm). The column effluent was monitored with diode array detector (KYN-365 nm, TRP-260 nm). The mobile phase was composed of 0.1 M acetic acid, 0.1 M ammonium acetate (pH 4.6) containing 1.8% of acetonitrile and it was pumped at a flow-rate of 0.2 mL/min. 3HK was measured as described by Heyes & Quearry (1988). The column effluent was monitored using a programmable electrochemical detector. Potential of the working electrode was 0.6 V. The mobile phase consisted of 0.1 M triethylamine, 0.1 M phosphoric acid, 0.3 mM EDTA, 8.2 mM heptane-1-sulfonic acid sodium salt, containing 2% of acetonitrile and was pumped at a flow-rate of 0.25 mL/min; 2  µL of the supernatant was injected into HPLC system for analysis.

### Statistical analysis

Shapiro–Wilk’s test of normality was used for data distribution analysis. The normally distributed data were shown as mean ± SD and analyzed using unpaired Student *t* test. The non-Gaussian data were presented as median (line) with interquartile range (box) and maximum and minimum values (whiskers) and analyzed using the non-parametric Mann–Whitney test. Spearman’s rank test calculated the correlations between study variables in 5/6 Nx rats. *P*-values less than 0.05 were considered statistically significant. The data were analyzed with Statistica version 12 computer software (StatSoft, Tulsa, OK, USA). Graphic design presentation of results was performed using R statistical software (version 3.3.2) or GraphPad Prism 6 (La Jolla, CA, USA).

## Results

### Animal characteristics

As shown in Table 1, the 5/6 Nx animals had elevated serum creatinine values and blood urea nitrogen, developed hyperparathyroidism and hypocalcemia. There were no differences in serum concentrations of phosphorus and serum activities of ALP and TRACP 5b between 5/6 Nx and controls. TRACP 5b activity showed only a trend to increase in 5/6 Nx animals compared to control animals.

**Table 1:** Body weight, biochemical parameters, and bone turnover biomarkers in sham-operated (Sham) and nephrectomized (5/6 Nx) rats.

	**Sham**	**5/6 Nx**
Final body weight, g	336.5 ± 63.8	313.6 ± 38.1
Creatinine, mg/dL	0.37 ± 0.07	0.63 ± 0.11***
Blood urea nitrogen, mg/dL	45.7 ± 6.1	77.7 ± 12.5***
Phosphorus, mg/dL	6.33 ± 1.83	5.87 ± 2.23
Calcium, mg/dL	5.86 ± 1.84	4.61 ± 1.54*
PTH, pg/mL	305.9 ± 91.2	526.4 ± 174.3***
ALP serum, U/L	45.8 ± 23.1	42.3 ± 19.1
TRACP 5b serum, U/L	158.0 ± 32.1	182.4 ± 47.6

**Notes.**

^∗^*p* < 0.05, ^∗∗∗^*p* < 0.001 vs sham group, unpaired Student *t* test. Data are mean ± SD, *n* = 15–18.

Final body weight weight at the time of sacrifice PTH parathyroid hormone ALP alkaline phosphatase TRACP 5b tartrate-resistant acid phosphatase form 5b

### Bone characteristics

Work to fracture, anterior-posterior periosteal diameter, wall thickness, cortical index, cross-sectional area, cross-sectional moment of inertia, mean relative wall thickness were significantly higher after nephrectomy compared to sham. There were no differences in Archimedes’ density between 5/6 Nx and controls (Table 2).

**Table 2:** Bone biomechanics, geometry, and bone mass density of the tibia in sham-operated (Sham) and nephrectomized (5/6 Nx) rats.

	Sham	5/6 Nx
***Bone biomechanics***		
Stiffness, N/mm	139.1 ± 45.9	165.0 ± 32.4
Yield load, N	58.8 ± 6.7	63.1 ± 8.4
Displacement at the yield load, µm	0.46 ± 0.12	0.40 ± 0.05
Ultimate load, N	77.7 ± 18.1	84.3 ± 12.0
Displacement at the ultimate load, µm	0.72 ± 0.17	0.76 ± 0.12
Work to fracture, mJ	29.3 ± 12.2	38.9 ± 9.0*
***Bone geometry***		
Tibial weight, mg	448.3 ± 73.5	477.4 ± 76.8
Tibial length, mm	35.1 ± 3.2	35.0 ± 2.9
Anterior-posterior periosteal diameter, mm	2.27 ± 0.12	2.37 ± 0.13*
Medial-lateral periosteal diameter, mm	3.32 ± 0.38	3.33 ± 0.19
Anterior-posterior endosteal diameter, mm	1.82 ± 0.15	1.77 ± 0.16
Medial-lateral endosteal diameter, mm	2.86 ± 0.35	2.79 ± 0.32
Wall thickness, mm	0.23 ± 0.04	0.29 ± 0.05***
Cortical index, % × 10^−3^	13.7 ± 1.9	17.1 ± 3.2***
Cross-sectional area, mm^2^	1.83 ± 0.4	2.31 ± 0.40**
Cross-sectional moment of inertia, mm^4^	1.07 ± 0.25	1.37 ± 0.32**
Mean relative wall thickness, × 10^−3^	0.21 ± 0.04	0.27 ± 0.06**
***Bone mass density***		
Archimedes’ density, g/cm^3^	1.48 ± 0.20	1.56 ± 0.22

**Notes.**

^∗^*p* < 0.05, ^∗∗^*p* < 0.01, ^∗∗∗^*p* < 0.001 vs sham group, unpaired Student *t* test. Data are mean ± SD *n* = 15–18.

### Kynurenine pathway metabolites in rat brain regions

**Figure 1:**
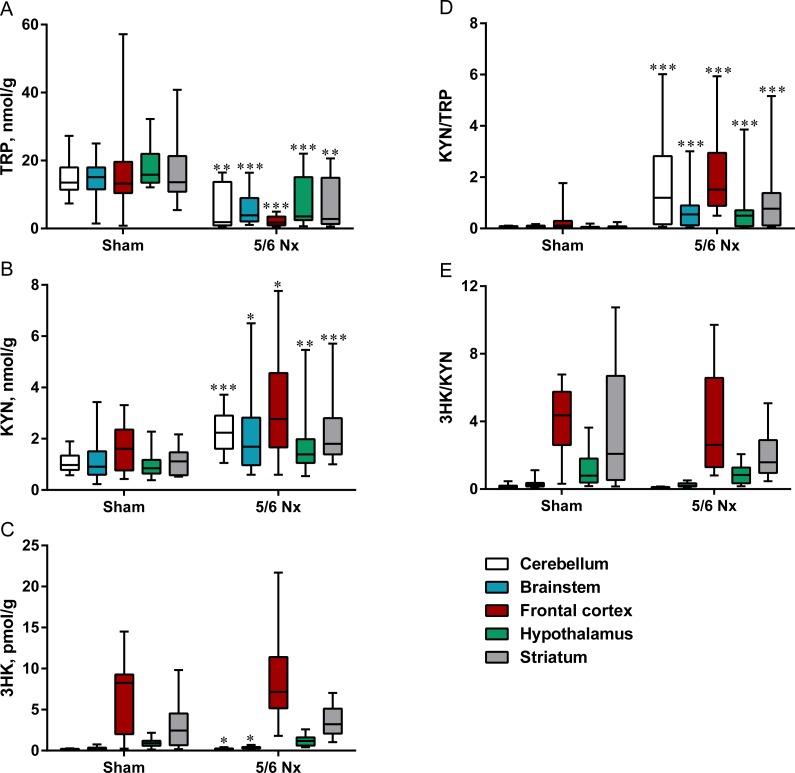
Tryptophan (TRP; A), kynurenine (KYN; B), 3-hydroxykynurenine (3HK; C) KYN/TRP ratio (D), and 3HK/KYN ratio (E) in different brain regions in sham-operated (Sham) and nephrectomized (5/6 Nx) rats. ^∗^*p* < 0.05, ^∗∗^*p* < 0.01, ^∗∗∗^*p* < 0.001 *vs* sham group, Mann–Whitney test. Results are shown as median (line) with interquartile range (box) and maximum and minimum values (whiskers).

Nephrectomized rats presented significantly lower TRP levels (Fig. 1A) and significantly higher KYN concentrations in all studied parts of the brain (Fig. 1B). Similarly to KYN levels, KYN/TRP ratio was significantly higher in all studied parts of the brain (Fig. 1D). Surgical resection of 5/6 kidney did not cause severe changes in the 3HK levels and 3-HKYN/KYN ratio in the brain (Figs. 1C and 1E).

### Relationships between kynurenine pathway metabolites in rat brain regions and bone properties in 5/6 Nx rats

**Figure 2:**
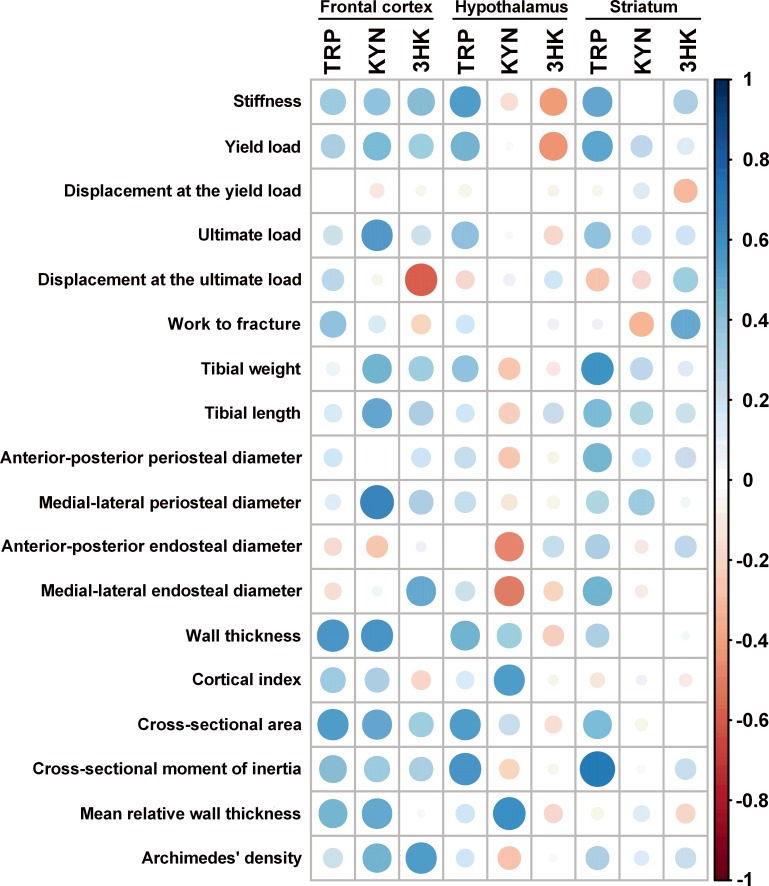
Spearman correlation matrix between tryptophan (TRP), kynurenine (KYN), and 3-hydroxykynurenine (3HK) in the frontal cortex, hypothalamus, and striatum and bone properties in 5/6 Nx rats. The intensity and size of color represent the strength of the correlation (darker and larger circles demonstrate the strong correlation). Blue colors, positive correlations; red colors, negative correlations.

KYN concentrations in the cerebellum correlated positively only with the medial-lateral periosteal diameter (Table S1). There were no statistically significant associations between kynurenine pathway metabolites in rat brainstem and main bone parameters (Table S2). KYN concentrations in the frontal cortex correlated positively with the ultimate load, tibial weight, tibial length, medial-lateral periosteal diameter, wall thickness, cross-sectional area, and mean relative wall thickness. TRP concentrations in the frontal cortex correlated positively with the wall thickness and cross-sectional area, while 3HK correlated positively with medial-lateral endosteal diameter and Archimedes’ density. 3HK concentrations in the frontal cortex also correlated negatively with displacement at the ultimate load (Fig. 2 and Table S3). There were also statistically significant positive correlations between TRP concentrations in the hypothalamus and stiffness, wall thickness, cross-sectional area, and cross-sectional moment of inertia. KYN levels in the hypothalamus correlated positively with the cortical index and mean relative wall thickness, but it was inversely associated with the medial-lateral periosteal diameter (Fig. 2 and Table S4). There were statistically significant positive correlations between TRP concentrations in the striatum and stiffness, yield load, tibial weight, medial-lateral endosteal diameter as well as the cross-sectional moment of inertia. 3HK concentrations in the striatum also correlated positively with work to fracture (Fig. 2 and Table S5).

## Discussion

In the present study, we found that not only serotonin but also kynurenines as tryptophan metabolites may be associated with the bone remodeling process. In contrast to complex mineral and hormone changes, central kynurenine metabolites seem to play a beneficial role in the development of bone changes in growing rats with experimentally induced CKD. We observed the intensified kynurenine turnover in all studied brain regions and the strongest positive relationships between KYN in the frontal cortex as well as TRP in the hypothalamus and striatum and bone biomechanical and geometrical parameters.

CKD was ranked 18th in the list of causes of a total number of global deaths in 2010 (annual death rate 16.3 per 100,000) (Lozano et al., 2013). In contrast to the clinically apparent advanced stage of CKD, precise calculation of the burden of less symptomatic or asymptomatic early stage of CKD, which accounts for 80–90% of all cases (Jha et al., 2013), is difficult. CKD-MBD is one of the most common and complex complications of CKD. In accordance with the definition, CKD-MBD occurs when the glomerular filtration is reduced by more than 40% (Moe et al., 2011). However, several results indicate that CKD-MBD may begin earlier in the disease process and clinically asymptomatic metabolic disturbances may precede the development of detectable abnormalities in plasma calcium, phosphorus, and parathyroid hormone (Pereira et al., 2009; Oliveira et al., 2010; Isakova et al., 2011; Sabbagh et al., 2012). Therefore, in our study, we used growing rats to understand the bone pathophysiology in the early stage of CKD. The animal model induced by subtotal nephrectomy mimics the progressive renal failure in humans and is commonly used to assess the pathophysiological aspects and the bone structure in the early CKD stages (Moscovici et al., 1996; Heveran et al., 2016).

We observed significant alterations in TRP and KYN concentrations as well as KYN/TRP ratio in all studied brain regions. Nephrectomized rats presented significantly lower TRP levels and significantly higher KYN levels. Observed alterations in the brain may be associated with the disturbances in circulating kynurenine pathway metabolites in the course of CKD (Saito et al., 2000; Pawlak et al., 2001a; Pawlak, Tankiewicz & Buczko, 2001b). Peripheral TRP, as well as KYN and 3HK, can enter the brain quite easily even under physiological conditions (Fukui et al., 1991; Pardridge, 1998). In the present study, a possible disruption of the blood–brain barrier under the pathological condition and increased permeation of kynurenine metabolites cannot be excluded due to the increased blood urea nitrogen in the serum and ongoing inflammation. Elevated concentrations of blood urea nitrogen are known to increase levels of reactive oxygen species (Zhang et al., 2004; D’Apolito et al., 2015), which are key mediators of blood–brain barrier breakdown (Pun, Lu & Moochhala, 2009). Mice with CKD induced by adenine feeding for four weeks presented higher serum concentrations of urea nitrogen. In this model, authors observed significant blood–brain barrier disruption and behavioral abnormalities (Mazumder et al., 2016). Blood–brain barrier disruption was also found in nephrectomized rats with chronic uremia and was linked to uremic encephalopathy (Jeppsson et al., 1982). On the other hand, approximately 60% of the KYN in the brain comes from the plasma; the remaining 40% is locally synthesized in the brain (Gal & Sherman, 1980). We have previously demonstrated the severe central kynurenine pathway disturbances in rats with end-stage of chronic renal insufficiency. In these animals, the levels of both KYN and 3HK were elevated in the different brain regions (Topczewska-Bruns et al., 2002). In our growing rats with early-stage of CKD, the activation of kynurenine pathway seems to be less pronounced.

Altered kynurenine pathway metabolites in certain brain regions correlated positively with the main parameters of bone biomechanics and bone geometry. Bone biomechanics were evaluated by the three-point bending test which is commonly used to measure the bone properties in rodents and other small animals (Goodyear & Aspden, 2012). The main biomechanical parameters include the stiffness, yield and ultimate loads and their corresponding displacements, and work to fracture. Surprisingly, work to fracture were significantly higher in nephrectomized rats than in controls, whereas yield and ultimate loads that determine the whole bone strength were similar between groups. These results suggest that the adaptive response in young rats could provide protection from the deleterious effects of the early stage of CKD on the bone strength. Similar effects on bone biomechanical parameters were observed in several studies on animals with the early stage of CKD (Iwamoto et al., 2012; Jokihaara et al., 2006; Heveran et al., 2016). In the recent study, Heveran et al. observed markedly altered maturation of bone material properties with distance from the periosteal surface in animals with moderate CKD induced by subtotal nephrectomy. These destructive alterations occurred despite minimal changes to bone microarchitecture and without differences in whole bone mechanical or material properties obtained from three-point bending test (Heveran et al., 2016). We observed the strongest positive relationships between KYN in the frontal cortex as well as TRP in the hypothalamus and striatum and bone biomechanical and geometrical parameters in growing rats with experimentally induced CKD. The relationships between above metabolites and geometrical parameters should be interpreted cautiously because geometrical analysis was calculated based on not very precise measurements with calipers. On the other hand, we obtained statistically significant and consistent results. Regulation of bone metabolism in multicellular organisms is the complex process. It depends on the interactions between different organs or tissues. Besides bone-resorbing osteoclasts and bone-forming osteoblasts, hypothalamic structures may be involved in the regulation of bone metabolism. The hypothalamus can act on bone metabolism through hormonal and neuronal signaling; leptin is one of the most extensively studied hormones that affects bone metabolism *via* a serotonin-hypothalamus pathway (Sharan & Yadav, 2014). Serotonergic neurons inhibit the synthesis of epinephrine and decrease sympathetic tone. The inhibition of sympathetic activity decreases signaling *via* the *β*_2_ adrenergic receptor in osteoblasts, which negatively affects osteoblast proliferation *via* the molecular clock gene/cyclin cascade and positively regulates bone resorption *via* protein kinase A/activating transcription factor 4-dependent pathway (Ducy & Karsenty, 2010). Our study confirms the important role of the hypothalamus in the bone regulation and suggests that the frontal cortex and striatum may also take part in the regulation of bone changes in CKD. The effect of kynurenines on bone metabolism, similarly to serotonin (Ducy & Karsenty, 2010), may be dependent on the site of their synthesis. In contrast to the serotonin, kynurenines can cross the blood–brain barrier, can accumulate in the brain during CKD, and can act on bone metabolism *via* both direct and indirect mechanism. In our study, there were no statistically significant associations between peripheral and central kynurenine pathway metabolites. It suggests that peripheral and central kynurenine pathways act as two separate systems on bone metabolism in the course of CKD. Further studies could confirm the protective effect of central kynurenines on bone metabolism and explain the mechanism of their action.

Our results suggest that in addition to the complex mineral and hormone changes such as hyperphosphatemia, hypocalcemia, hyperparathyroidism, and active vitamin D deficiency, kynurenine pathway metabolites may play an important role in the development of CKD-MBD. Studying the bone regulation by kynurenines has brought to light on the pleiotropic nature of these molecules. Our results have enriched the understanding of the pathophysiology of CKD-MBD. The effect of kynurenines on bone metabolism may be closely dependent on the site of their occurrence. Peripheral and central kynurenines may exert opposite influences on the bone formation. Among all studied brain regions, intensified kynurenine turnover in the frontal cortex, hypothalamus, and striatum may be especially responsible for the bone disturbances in CKD. The present study for the first time demonstrates the association between intensified central kynurenine turnover and bone metabolism in growing rats with CKD. The observed results open new possibilities for the prevention, diagnosis, and treatment of bone abnormalities in CKD patients.

##  Supplemental Information

10.7717/peerj.3199/supp-1 Table S1 The association between tryptophan (TRP), kynurenine (KYN), and 3-hydroxykynurenine (3HK) concentrations in the cerebellum and bone properties in 5/6 Nx rats NS, not significant.

10.7717/peerj.3199/supp-2 Table S2 The association between tryptophan (TRP), kynurenine (KYN), and 3-hydroxykynurenine (3HK) concentrations in the brainstem and bone properties in 5/6 Nx rats NS, not significant.

10.7717/peerj.3199/supp-3 Table S3 The association between tryptophan (TRP), kynurenine (KYN), and 3-hydroxykynurenine (3HK) concentrations in the frontal cortex and bone properties in 5/6 Nx rats NS, not significant.

10.7717/peerj.3199/supp-4 Table S4 The association between tryptophan (TRP), kynurenine (KYN), and 3-hydroxykynurenine (3HK) concentrations in the hypothalamus and bone properties in 5/6 Nx rats NS, not significant.

10.7717/peerj.3199/supp-5 Table S5 The association between tryptophan (TRP), kynurenine (KYN), and 3-hydroxykynurenine (3HK) concentrations in the striatum and bone properties in 5/6 Nx rats NS, not significant.

10.7717/peerj.3199/supp-6 Data S1 Raw data

## Additional Information and Declarations

### Data Availability

The following information was supplied regarding data availability: The raw data has been supplied as Data S1.
