# Supplementary material for: A link between central kynurenine metabolism and bone strength in rats with chronic kidney disease
Source: PeerJ. 2017 Apr 20;5:e3199. doi: 10.7717/peerj.3199 (PMC5401623; doi:10.7717/peerj.3199)
Supplement: Table S1 — NS, not significant. [file peerj-05-3199-s001.docx]

|  | TRP | KYN | 3HK |
| --- | --- | --- | --- |
| *Bone biomechanics* | | | |
| Stiffness | r = 0.254  NS | r = 0.155  NS | r = 0.030  NS |
| Yield load | r = 0.372  NS | r = 0.351  NS | r = 0.098  NS |
| Displacement at the yield load | r = 0.152  NS | r = 0.047  NS | r = 0.003  NS |
| Ultimate load | r = 0.123  NS | r = 0.423  NS | r = 0.199  NS |
| Displacement at the ultimate load | r = -0.319  NS | r = -0.135  NS | r = 0.024  NS |
| Work to fracture | r = -0.185  NS | r = -0.102  NS | r = -0.190  NS |
| *Bone geometry* | | | |
| Tibial weight | r = 0.287  NS | r = 0.295  NS | r = 0.185  NS |
| Tibial length | r = 0.154  NS | r = 0.149  NS | r = 0.193  NS |
| Anterior-posterior periosteal diameter | r = 0.287  NS | r = -0.193  NS | r = 0.160  NS |
| Medial-lateral periosteal diameter | r = 0.086  NS | r = 0.514  p = 0.029 | r = 0.294  NS |
| Anterior-posterior endosteal diameter | r = 0.223  NS | r = -0.419  NS | r = -0.172  NS |
| Medial-lateral endosteal diameter | r = 0.384  NS | r = -0.154  NS | r = -0.167  NS |
| Wall thickness | r = 0.182  NS | r = 0.318  NS | r = 0.108  NS |
| Cortical index | r = -0.168  NS | r = 0.315  NS | r = 0.229  NS |
| Cross-sectional area | r = 0.291  NS | r = 0.118  NS | r = 0.034  NS |
| Cross-sectional moment  of inertia | r = 0.372  NS | r = -0.067  NS | r = 0.003  NS |
| Mean relative wall thickness | r = -0.072  NS | r = 0.388  NS | r = 0.290  NS |
| *Bone mass density* | | | |
| Archimedes’ density | r = 0.158  NS | r = 0.048  NS | r = 0.216  NS |

**Table S1.** The association between tryptophan (TRP), kynurenine (KYN), and 3-hydroxykynurenine (3HK) concentrations in the cerebellum and bone properties in 5/6 Nx rats.

NS, not significant.
