# Supplementary material for: A link between central kynurenine metabolism and bone strength in rats with chronic kidney disease
Source: PeerJ. 2017 Apr 20;5:e3199. doi: 10.7717/peerj.3199 (PMC5401623; doi:10.7717/peerj.3199)
Supplement: Table S2 — NS, not significant. [file peerj-05-3199-s002.docx]

**Table S2.** The association between tryptophan (TRP), kynurenine (KYN), and 3-hydroxykynurenine (3HK) concentrations in the brainstem and bone properties in 5/6 Nx rats.

|  | TRP | KYN | 3HK |
| --- | --- | --- | --- |
| *Bone biomechanics* | | | |
| Stiffness | r = 0.209  NS | r = -0.115  NS | r = -0.084  NS |
| Yield load | r = 0.267  NS | r = 0.001  NS | r = -0.327  NS |
| Displacement at the yield load | r = 0.176  NS | r = 0.009  NS | r = -0.080  NS |
| Ultimate load | r = 0.113  NS | r = 0.100  NS | r = -0.156  NS |
| Displacement at the ultimate load | r = -0.133  NS | r = -0.035  NS | r = 0.173  NS |
| Work to fracture | r = -0.148  NS | r = -0.186  NS | r = 0.017  NS |
| *Bone geometry* | | | |
| Tibial weight | r = 0.283  NS | r = 0.064  NS | r = -0.102  NS |
| Tibial length | r = 0.288  NS | r = 0.230  NS | r = -0.084  NS |
| Anterior-posterior periosteal diameter | r = 0.250  NS | r = -0.337  NS | r = -0.315  NS |
| Medial-lateral periosteal diameter | r = 0.154  NS | r = -0.364  NS | r = -0.105  NS |
| Anterior-posterior endosteal diameter | r = 0.285  NS | r = -0.342  NS | r = -0.467  NS |
| Medial-lateral endosteal diameter | r = 0.115  NS | r = -0.391  NS | r = -0.226  NS |
| Wall thickness | r = 0.128  NS | r = 0.081  NS | r = -0.061  NS |
| Cortical index | r = -0.025  NS | r = 0.320  NS | r = 0.205  NS |
| Cross-sectional area | r = 0.205  NS | r = -0.038  NS | r = -0.195  NS |
| Cross-sectional moment  of inertia | r = 0.368  NS | r = -0.319  NS | r = -0.387  NS |
| Mean relative wall thickness | r = -0.092  NS | r = 0.331  NS | r = 0.209  NS |
| *Bone mass density* | | | |
| Archimedes’ density | r = 0.152  NS | r = -0.003  NS | r = 0.106  NS |

NS, not significant.
