# Supplementary material for: A link between central kynurenine metabolism and bone strength in rats with chronic kidney disease
Source: PeerJ. 2017 Apr 20;5:e3199. doi: 10.7717/peerj.3199 (PMC5401623; doi:10.7717/peerj.3199)
Supplement: Table S3 — NS, not significant. [file peerj-05-3199-s003.docx]

**Table S3.** The association between tryptophan (TRP), kynurenine (KYN), and 3-hydroxykynurenine (3HK) concentrations in the frontal cortex and bone properties in 5/6 Nx rats.

|  | TRP | KYN | 3HK |
| --- | --- | --- | --- |
| *Bone biomechanics* | | | |
| Stiffness | r = 0.366  NS | r = 0.391  NS | r = 0.412  NS |
| Yield load | r = 0.315  NS | r = 0.432  NS | r = 0.338  NS |
| Displacement at the yield load | r = 0.001  NS | r = -0.107  NS | r = -0.053  NS |
| Ultimate load | r = 0.203  NS | r = 0.542  p = 0.020 | r = 0.203  NS |
| Displacement at the ultimate load | r = 0.263  NS | r = -0.057  NS | r = -0.577  p = 0.012 |
| Work to fracture | r = 0.386  NS | r = 0.153  NS | r = -0.206  NS |
| *Bone geometry* | | | |
| Tibial weight | r = 0.091  NS | r = 0.474  p = 0.047 | r = 0.338  NS |
| Tibial length | r = 0.166  NS | r = 0.492  p = 0.038 | r = 0.321  NS |
| Anterior-posterior periosteal diameter | r = 0.174  NS | r = 0.021  NS | r = 0.198  NS |
| Medial-lateral periosteal diameter | r = 0.128  NS | r = 0.641  p = 0.004 | r = 0.324  NS |
| Anterior-posterior endosteal diameter | r = -0.165  NS | r = -0.255  NS | r = 0.052  NS |
| Medial-lateral endosteal diameter | r = -0.152  NS | r = 0.045  NS | r = 0.487  p = 0.040 |
| Wall thickness | r = 0.569  p = 0.014 | r = 0.571  p = 0.013 | r = 0.027  NS |
| Cortical index | r = 0.350  NS | r = 0.324  NS | r = -0.196  NS |
| Cross-sectional area | r = 0.536  p = 0.022 | r = 0.492  p = 0.038 | r = 0.335  NS |
| Cross-sectional moment  of inertia | r = 0.414  NS | r = 0.368  NS | r = 0.315  NS |
| Mean relative wall thickness | r = 0.455  NS | r = 0.509  p = 0.031 | r = -0.028  NS |
| *Bone mass density* | | | |
| Archimedes’ density | r = 0.207  NS | r = 0.461  NS | r = 0.538  p = 0.021 |

NS, not significant.
