# Supplementary material for: A link between central kynurenine metabolism and bone strength in rats with chronic kidney disease
Source: PeerJ. 2017 Apr 20;5:e3199. doi: 10.7717/peerj.3199 (PMC5401623; doi:10.7717/peerj.3199)
Supplement: Table S4 — NS, not significant. [file peerj-05-3199-s004.docx]

**Table S4.** The association between tryptophan (TRP), kynurenine (KYN), and 3-hydroxykynurenine (3HK) concentrations in the hypothalamus and bone properties in 5/6 Nx rats.

|  | TRP | KYN | 3HK |
| --- | --- | --- | --- |
| *Bone biomechanics* | | | |
| Stiffness | r = 0.532  p = 0.023 | r = -0.156  NS | r = -0.414  NS |
| Yield load | r = 0.461  NS | r = -0.026  NS | r = -0.441  NS |
| Displacement at the yield load | r = -0.076  NS | r = 0.012  NS | r = -0.061  NS |
| Ultimate load | r = 0.408  NS | r = -0.025  NS | r = -0.189  NS |
| Displacement at the ultimate load | r = -0.178  NS | r = 0.067  NS | r = 0.173  NS |
| Work to fracture | r = 0.182  NS | r = 0.013  NS | r = 0.061  NS |
| *Bone geometry* | | | |
| Tibial weight | r = 0.383  NS | r = -0.255  NS | r = -0.094  NS |
| Tibial length | r = 0.174  NS | r = -0.236  NS | r = 0.226  NS |
| Anterior-posterior periosteal diameter | r = 0.243  NS | r = -0.246  NS | r = -0.063  NS |
| Medial-lateral periosteal diameter | r = 0.240  NS | r = -0.133  NS | r = -0.078  NS |
| Anterior-posterior endosteal diameter | r = 0.014  NS | r = -0.465  NS | r = 0.243  NS |
| Medial-lateral endosteal diameter | r = 0.203  NS | r = -0.493  p = 0.038 | r = -0.202  NS |
| Wall thickness | r = 0.477  p = 0.045 | r = 0.339  NS | r = -0.237  NS |
| Cortical index | r = 0.165  NS | r = 0.531  p = 0.023 | r = -0.051  NS |
| Cross-sectional area | r = 0.531  p = 0.023 | r = 0.235  NS | r = -0.158  NS |
| Cross-sectional moment  of inertia | r = 0.571  p = 0.023 | r = -0.214  NS | r = -0.051  NS |
| Mean relative wall thickness | r = 0.189  NS | r = 0.594  p = 0.009 | r = -0.185  NS |
| *Bone mass density* | | | |
| Archimedes’ density | r = 0.174  NS | r = -0.289  NS | r = -0.030  NS |

NS, not significant.
