# Supplementary material for: A link between central kynurenine metabolism and bone strength in rats with chronic kidney disease
Source: PeerJ. 2017 Apr 20;5:e3199. doi: 10.7717/peerj.3199 (PMC5401623; doi:10.7717/peerj.3199)
Supplement: Table S5 — NS, not significant. [file peerj-05-3199-s005.docx]

**Table S5.** The association between tryptophan (TRP), kynurenine (KYN), and 3-hydroxykynurenine (3HK) concentrations in the striatum and bone properties in 5/6 Nx rats.

|  | TRP | KYN | 3HK |
| --- | --- | --- | --- |
| *Bone biomechanics* | | | |
| Stiffness | r = 0.490  p = 0.039 | r = -0.003  NS | r = 0.320  NS |
| Yield load | r = 0.517  p = 0.028 | r = 0.255  NS | r = 0.148  NS |
| Displacement at the yield load | r = -0.052  NS | r = 0.134  NS | r = -0.303  NS |
| Ultimate load | r = 0.385  NS | r = 0.197  NS | r = 0.195  NS |
| Displacement at the ultimate load | r = -0.284  NS | r = -0.177  NS | r = 0.338  NS |
| Work to fracture | r = 0.052  NS | r = -0.325  NS | r = 0.483  p = 0.042 |
| *Bone geometry* | | | |
| Tibial weight | r = 0.585  p = 0.011 | r = 0.272  NS | r = 0.114  NS |
| Tibial length | r = 0.435  NS | r = 0.288  NS | r = 0.205  NS |
| Anterior-posterior periosteal diameter | r = 0.458  NS | r = 0.178  NS | r = 0.228  NS |
| Medial-lateral periosteal diameter | r = 0.282  NS | r = 0.368  NS | r = 0.047  NS |
| Anterior-posterior endosteal diameter | r = 0.329  NS | r = -0.091  NS | r = 0.253  NS |
| Medial-lateral endosteal diameter | r = 0.471  p = 0.048 | r = -0.081  NS | r = -0.002  NS |
| Wall thickness | r = 0.302  NS | r = 0.011  NS | r = 0.034  NS |
| Cortical index | r = -0.113  NS | r = 0.051  NS | r = -0.086  NS |
| Cross-sectional area | r = 0.449  NS | r = -0.075  NS | r = -0.007  NS |
| Cross-sectional moment  of inertia | r = 0.701  p = 0.001 | r = -0.038  NS | r = 0.238  NS |
| Mean relative wall thickness | r = -0.073  NS | r = 0.141  NS | r = -0.199  NS |
| *Bone mass density* | | | |
| Archimedes’ density | r = 0.302  NS | r = 0.110  NS | r = 0.230  NS |

NS, not significant.
